# De novo *CDKN1C* variant in Beckwith–Wiedermann spectrum with atypical complications

**DOI:** 10.1038/s41439-025-00316-0

**Published:** 2025-05-28

**Authors:** Yuri Moriura, Yosuke Nishio, Shintaro Ichimura, Haruka Noda, Yoshihiro Tanahashi, Hikaru Yamamoto, Yuka Nakazawa, Taichi Oso, Yoshiaki Sato, Toshiki Takenouchi, Shinji Saitoh, Yukako Muramatsu, Tomoo Ogi

**Affiliations:** 1 Department of Pediatrics, Nagoya University Graduate School of Medicine, Nagoya, Japan; 2 Department of Genetics, Research Institute of Environmental Medicine, Nagoya University, Nagoya, Japan; 3 Department of Human Genetics and Molecular Biology, Nagoya University Graduate School of Medicine, Nagoya, Japan; 4 Division of Neonatology, Center for Maternal-Neonatal Care, Nagoya University Hospital, Nagoya, Japan; 5 Department of Pediatrics, TOYOTA Memorial Hospital, Toyota, Japan; 6 Department of Pediatrics, Keio University School of Medicine, Tokyo, Japan; 7 Department of Pediatrics and Neonatology, Nagoya City University Graduate School of Medical Sciences, Nagoya, Japan

**Keywords:** Imprinting, Next-generation sequencing, DNA methylation

## Abstract

Beckwith–Wiedemann spectrum (BWSp) is a genomic imprinting disorder characterized by a wide range of clinical features. Here we report an infant with BWSp and atypical features, for whom long-read sequencing confirmed a de novo *CDKN1C* variant that occurred on the maternally inherited allele and excluded other genetic etiologies. These findings not only expand the BWSp concept but also highlight the potential value of allelic origin analysis in cases with atypical presentations.

Beckwith–Wiedemann spectrum (BWSp) encompasses a range of clinical manifestations caused by genetic and epigenetic alterations at chromosome 11p15.5. Patients with a clinical diagnosis of Beckwith–Wiedemann syndrome with or without an (epi)genetic change, those with atypical Beckwith–Wiedemann syndrome with fewer cardinal features and an (epi)genetic change and those with isolated lateralized overgrowth with an (epi)genetic change are all classified under BWSp^1,2^.

The most common (epi)genetic alterations are loss of methylation at the maternal KCNQ1OT1:TSS-DMR, followed by paternal uniparental disomy of 11p15.5 and increased methylation at the maternal H19/IGF2:IG-DMR^3^. Point mutations of *CDKN1C* (OMIM#600856) are relatively rare, contributing to less than 10% of all cases, and are even rarer in sporadic cases than in familial ones^4^. The paternal *CDKN1C* is transcriptionally silenced via hypomethylation at KCNQ1OT1:TSS-DMR, with nearly all transcripts derived from the maternal allele^5^, meaning that pathogenic variants in *CDKN1C* must occur on the maternal allele. Despite this molecular background, determining the allelic origin is not routinely performed in clinical practice, even in cases with atypical complications.

In this study, we report a case of BWSp in an infant presenting with cholestasis and intestinal malrotation. Long-read sequencing determined the allelic origin of a de novo *CDKN1C* variant and excluded potential dual molecular diagnoses, highlighting the potential utility of long-read sequencing technologies. The experimental protocol was approved by the Committee for Ethical Issues at Nagoya University School of Medicine (2021-016321939 and 2022-011225810). Written informed consent, including consent for the use of photographs, was obtained from the patient and guardians. Detailed clinical descriptions are summarized in the Supplementary Information. In brief, a 3-month-old girl (Fig. 1a) was born at 30 weeks of gestation with a body weight of 1088 g (−1.5 s.d.) and length of 37.5 cm (−0.9 s.d.). She presented with macroglossia, a nevus simplex on the forehead, bilateral inguinal hernias and recurrent hypoglycemia without elevated insulin levels, findings indicative of BWSp (Fig. 1b, c). Her BWSp score was 5, fulfilling the diagnostic criteria for classical BWS. However, the presence of several uncommon complications—specifically, intestinal malrotation requiring prolonged total parenteral nutrition (TPN) (Fig. 1c,d) and cholestasis (Fig. 1d)—resulted in an overall atypical clinical presentation.

**Fig. 1:**
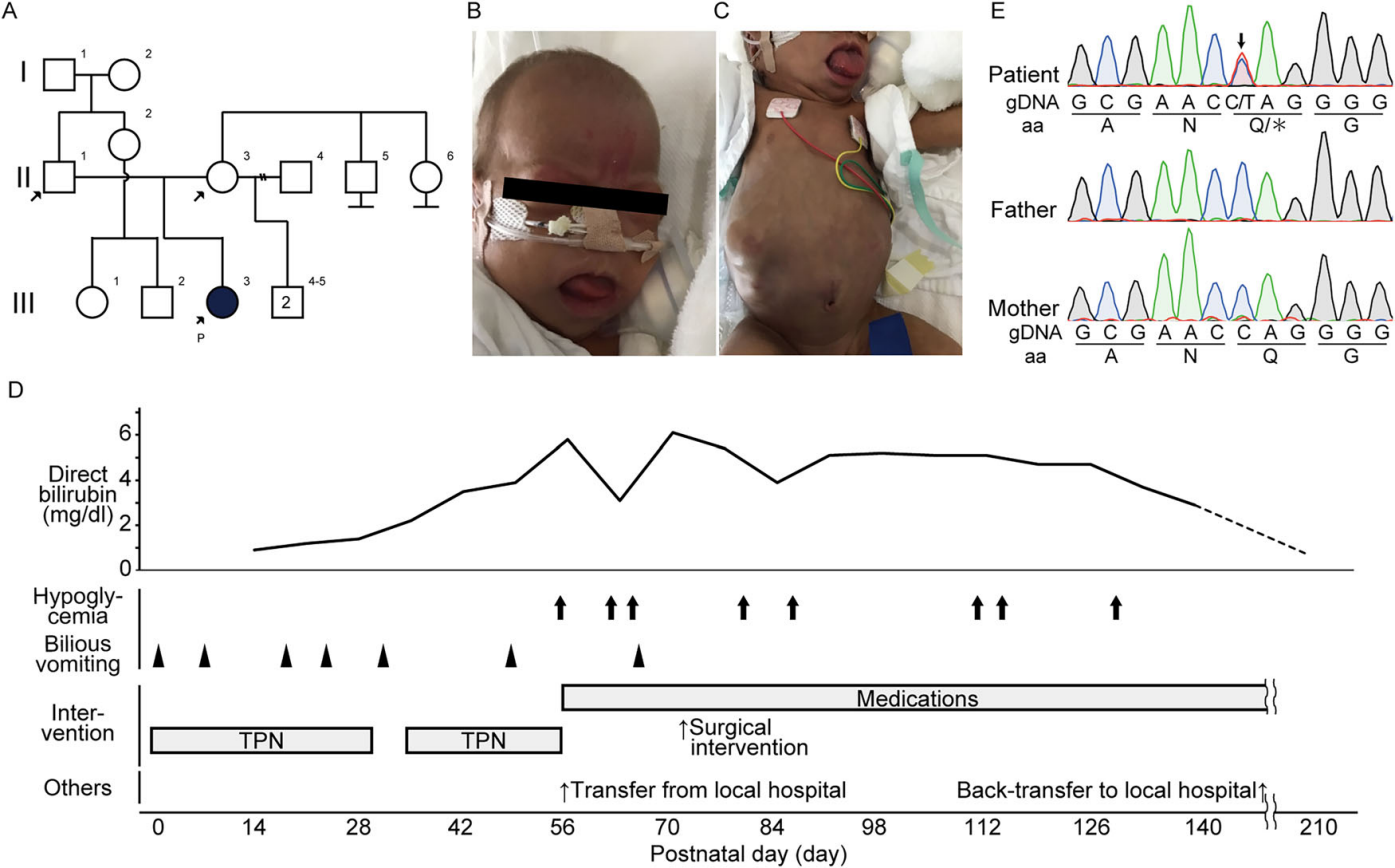
Clinical features and genetic analysis of the patient. **A** Pedigree analysis of the patient. The clients are indicated with arrow, and the proband is filled with black and indicated with the letter ‘P’. **B**, **C**, Representative pictures of macroglossia, nevus simplex (**B**) and abdominal distention (**C**) during the neonatal period. **D** The clinical course of the patient. The bold arrow and arrowhead indicate the episodes of hypoglycemia and bilious vomiting, respectively. **E** Sanger sequence. The arrow indicates the base substitutions, resulting in a Q (glutamine) to asterisk (stop codon) substitution in a heterozygous state.

Exome sequencing identified a de novo nonsense variant in *CDKN1C*, NM_001122630.2:c.655C>T, p.(Gln219*) (Fig. 1e) but no genetic candidates to explain the expanded phenotypes beyond BWSp. The variant was absent from both the gnomAD v4.1.0 and ToMMo 38KJPN databases but was recorded in ClinVar (RCV001218456). Based on the American College of Medical Genetics (ACMG) guidelines, the variant was classified as pathogenic (PVS1, PS2-moderate and PM2-supporting)^6^. However, the diagnosis remained inconclusive due to uncertainty regarding the allelic origin of the variant.

Considering the atypical presentation, we conducted long-read sequencing to address this uncertainty, which revealed no additional genetic candidates. Notably, haplotype phasing and methylation profiling confirmed that the variant was located on haplotype 1, where hypermethylation at KCNQ1OT1:TSS-DMR was observed, confirming it was on the maternal allele (Fig. 2). Although the methylation profile at H19/IGF2:IG-DMR could not be fully assessed due to insufficient depth, the presence of hyper- and hypomethylation at differentially methylated regions, IGF2:Ex9-DMR, of *IGF2* (Supplementary Figure) further supported that the variant was on the maternally inherited allele^7^.

**Fig. 2:**
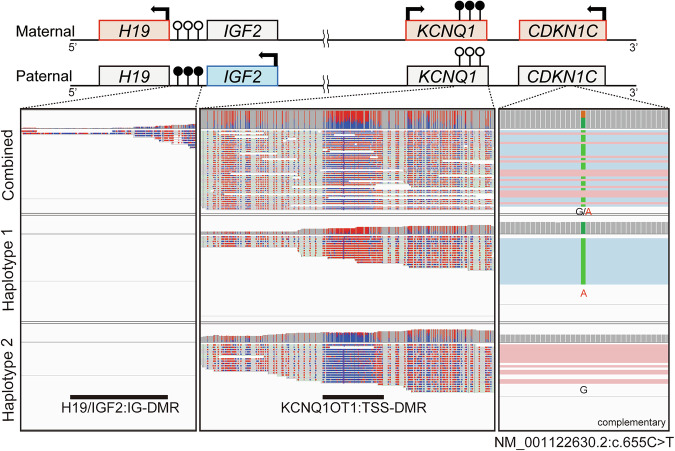
Methylation profile of imprinting control regions at 11p15.5. Top: a schematic illustration of an 11p15.5 imprinting cluster with a physiological methylation profile. The squares filled with red and blue with bold arrows indicate genes transcribed maternally and paternally, respectively. The squares filled with gray indicate the ones that are not transcribed from each allele. The circles with vertical lines indicate the methylation status, where the circles filled with black indicate methylated regions, whereas the unfilled circles indicate unmethylated regions. Bottom: the mapping data with the methylation profile of the patient visualized with Integrative Genomics Viewer (IGV), coloring alignment by the methylation profile, 5-methylcytosine (5mC). The left panel extracts regions surrounding H19/IGF2:IG-DMR (GRCh38: chr11:1997582-2003510), the center panel extracts the KCNQ1OT1:TSS-DMR (GRCh38: chr11:2698718-2701029) and the right panel extracts the region around the variant (GRCh38:chr11:2884786 to 2884819). Methylation status is colored from red to blue, indicating hyper- to hypomethylation of the bases.

BWSp is a disorder with a broad clinical spectrum. Clinicians sometimes need to consider differential diagnoses or the possibility of a dual molecular diagnosis when patients lack the typical phenotypes or present with a variety of atypical complications^8^.

Cholestasis has not been directly reported as a typical complication of BWSp. Similarly, while described in rare cases of BWSp, intestinal malrotation remains an exceedingly rare and not well-established feature of the syndrome’s phenotype^9,10^. Consequently, these atypical features necessitated ruling out dual molecular diagnoses and exploring other potential genetic etiologies, such as Alagille syndrome and neonatal intrahepatic cholestasis caused by citrine deficiency, and confirming the molecular diagnosis of BWSp. When dealing with imprinting disorders, determining the allelic origin could be important for establishing pathogenicity. Nevertheless, the allelic origin is not routinely determined, and de novo variants are often presumed to be pathogenic (Supplementary Table). In fact, in HGMD Professional 2024.1, among the 57 documented point mutations with inheritance information, 14% were classified as de novo. However, none of these cases had been further investigated to confirm the allelic origin.

In this case, we successfully used long-read sequencing to accurately determine the maternal origin of the de novo *CDKN1C* variant and exclude potential dual molecular diagnoses, although there are potential limitations. First, DNA methylation testing was not performed. This decision was driven by the need for broader genetic analysis to rule out non-BWSp causes with immediate interventional implications in the neonatal period. Second, while advanced techniques such as long-read sequencing offer a notable diagnostic precision, their broader application is often limited by cost and accessibility. Finally, atypical complications may be attributed to prematurity, prolonged TPN or idiopathic causes.

In conclusion, cholestasis and intestinal malrotation complicated the patient’s clinical presentation as BWSp. Advanced genetic analysis using long-read sequencing enabled the accurate molecular diagnosis by determining the parental origin of the *CDKN1C* variant and ruling out other potential genetic causes and could have contributed novel insights into the phenotypic spectrum of BWSp.

## HGV database

The relevant data from this Data Report are hosted at the Human Genome Variation Database at 10.6084/m9.figshare.hgv.3509.

## Supplementary information

Supplementary Information including detailed case presentations, supplementary methods, figures, table and references.

## Data Availability

The *CDKN1C* variant identified in this study has been deposited to ClinVar under the accession ID VCV000132861.8 (https://www.ncbi.nlm.nih.gov/clinvar/variation/VCV000132861.8/?redir=vcv) and is publicly available for reference. The other data that support the findings of this study are available on request. The data are not publicly available due to privacy or ethical restrictions.
