# Supplementary material for: De novo CDKN1C variant in Beckwith–Wiedermann spectrum with atypical complications
Source: Hum Genome Var. 2025 May 28;12:9. doi: 10.1038/s41439-025-00316-0 (PMC12120123; doi:10.1038/s41439-025-00316-0)
Supplement: Supplementary file 1 — Supplementary Information including detailed case presentations, supplementary methods, figures, table and references. [file 41439_2025_316_MOESM1_ESM.docx]

**Case**

The patient, a 3-month-old girl without any remarkable family history (Figure 1A), was born at 30 weeks of gestation due to maternal preeclampsia. Her mother and father were 42 and 36 years old, respectively, and the pregnancy was established with in vitro fertilization, or intracytoplasmic sperm injection. She had an Apgar score of 1 at 1 minute and 2 at 5 minutes, with body weight was 1088 g (-1.5 SD), length 37.5 cm (-0.9 SD), and head circumference 24 cm (-1.8 SD) at birth. She exhibited macroglossia, nevus simplex on the forehead, and bilateral inguinal hernias, alongside complications associated with preterm birth, indicative of BWS (Figure 1B and C). In addition to typical BWS phenotypes, she had a variety of complications, including recurrent bilious vomiting and abdominal retention, requiring prolonged total parenteral nutrition (TPN) (Figure 1C), subsequently diagnosed as intestinal malrotation requiring surgical intervention, and cholestasis starting as early as 2 weeks of life, persisting beyond 5 months despite interventions with deoxycholic acid and monoammonium glycyrrhizinate / glycine / DL-methionine (Figure 1D). Recurrent hypoglycemia without an elevated insulin level was also documented due to the inconclusive cause, BWS phenotype or malnutrition, that was managed with frequent feedings and supplementation with medium-chain triglyceride (MCT) oil. In terms of growth parameters, she never showed an overgrowth phenotype during her infancy, with body weight was 1769 g (-3.1 SD), length 43 cm (-2.4 SD), and head circumference 30 cm (-2.2 SD) at the age of 56 days, i.e., the corrected gestational age of 38 weeks. Given varieties of phenotypes that cannot be explained by the single etiology, BWS, exome sequencing (ES) was performed for the genetic diagnosis explaining phenotypes atypical for BWS, especially cholestasis, for which the genetic diagnosis could potentially help clinicians to choose the best intervention.

**Supplementary Methods**

**Exome sequencing**

Exome sequencing was performed as described previously (Otsuji et al., 2022). Briefly, genomic DNA was extracted form peripheral blood with QIAamp DNA Blood Midi Kit (QIAGEN) according to the manufacturer’s instruction, partitioned using the SureSelect XT Human All Exon V6 capture library (Agilent Technologies) or Twist Comprehensive Exome Panel (Twist Bioscience), followed by DNA sequencing performed with a HiSeq 4000 or NovaSeq 6000 (Illumina) sequencer. After mapping to the reference genome (hg19), we excluded variants with a minor allele frequency over 1 % in public databases, except previously identified pathogenic variants in the ClinVar database and the Human Gene Mutation Database. We then focused on nonsynonymous single nucleotide variants, insertions and deletions, and splice site variants. Identified variants were confirmed by Sanger sequencing of PCR-amplified products with the following primers: *CDKN1C* (forward: 5’- TTGATCGCCGCGCCGTT-3’; reverse: 5’- TCTACCGCGAGACGGTGCA-3’).

**Long-read sequencing**

DNA samples were sheared using the Megaruptor 3 system, and library preparation was performed with the SMRTbell prep kit 3.0. HiFi reads were generated on the Revio system and aligned to the GRCh38 reference genome with pbmm2 (v1.10.0). Haplotype-phasing, methylation profiling, and structural variation calling were conducted using HiPhase (v1.4.2), MethBat (v.0.11.0), and pbsv (v2.9.0), respectively.

The *CDKN1C* gene sequence used in this study was based on the GenBank reference sequence NM_001122630.2.

**Databases**

The following databases were used: gnomAD v4.1.0 (https://gnomad.broadinstitute.org/); ToMMo 38KJPN Allele Frequency Panel (https://jmorp.megabank.tohoku.ac.jp/); HGMD Professional 2024.1 (<https://www.hgmd.cf.ac.uk/>).

**Supplementary Figure**


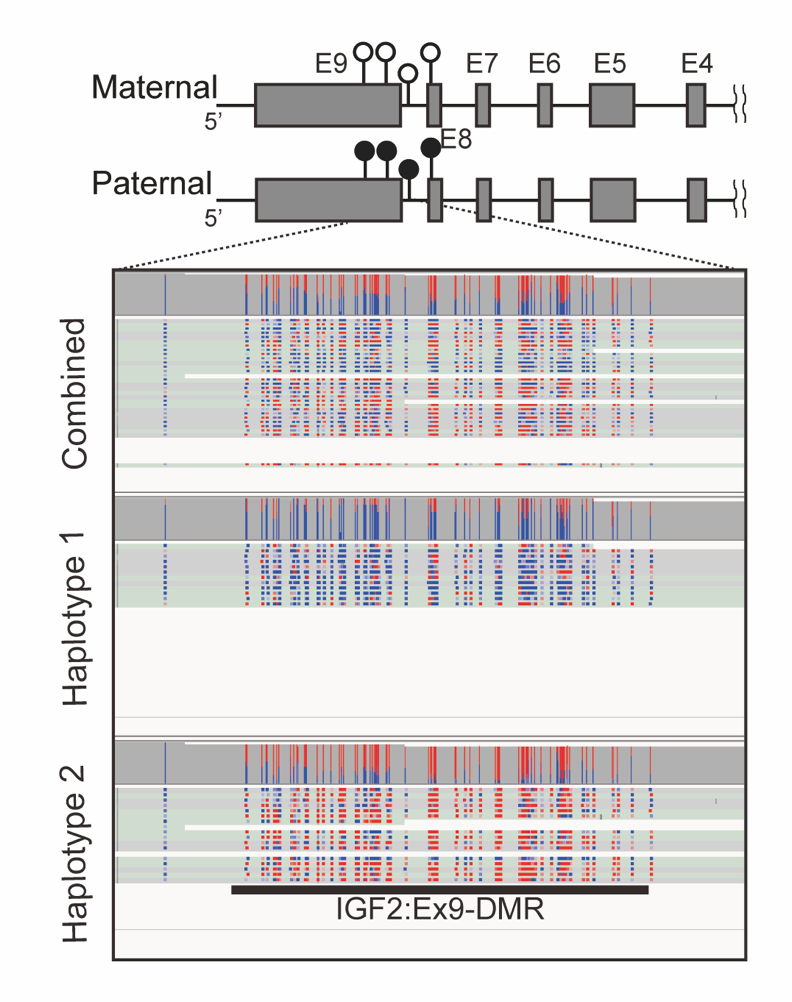
Supplementary Figure. Methylation profile at differentiated methylation region of *IGF2*
Upper panel indicates the schematic illustration of *IGF2* with physiological methylation profile. The squares indicate exons, and the circles with a vertical line indicate the methylation status. The circles filled with black indicate methylated region, whereas the unfilled circles unmethylated one. Lower panel indicates the mapping data around IGF2:Ex9-DMR (GRCh38: chr11:2132761-2133882) with methylation profile of the patient visualized with Integrative Genomics Viewer (IGV), coloring alignment by the methylation profile, 5-methylcytosine (5mC). Methylation status is colored from red to blue, indicating hyper- to hypomethylation of the bases.

**Supplementary Table**

Supplementary Table. List of all point mutations (missense/nonsense, splicing, small insertions, and small indels) with available inheritance information in HGMD Professional 2024.1.

**Supplementary References**

Best, L. G., Duffy, K. A., George, A. M., Ganguly, A., & Kalish, J. M. (2023). Familial
Beckwith-Wiedemann syndrome in a multigenerational family: Forty years of careful phenotyping. *Am J Med Genet A*, *191*(2), 348-356. <https://doi.org/10.1002/ajmg.a.63026>

Cardoso, L. C. A., Parra, A., Gil, C. R., Arias, P., Gallego, N., Romanelli, V.,…Lapunzina, P. (2022). Clinical Spectrum and Tumour Risk Analysis in Patients with Beckwith-Wiedemann Syndrome Due to CDKN1C Pathogenic Variants. *Cancers (Basel)*, *14*(15). <https://doi.org/10.3390/cancers14153807>

Engel, J. R., Smallwood, A., Harper, A., Higgins, M. J., Oshimura, M., Reik, W.,…Maher, E. R. (2000). Epigenotype-phenotype correlations in Beckwith-Wiedemann syndrome. *J Med Genet*, *37*(12), 921-926. <https://doi.org/10.1136/jmg.37.12.921>

Gorukmez, O., Gorukmez, O., & Topak, A. (2023). Clinical exome sequencing findings in 1589 patients. *Am J Med Genet A*, *191*(6), 1557-1564. <https://doi.org/10.1002/ajmg.a.63190>

Hatada, I., Nabetani, A., Morisaki, H., Xin, Z., Ohishi, S., Tonoki, H.,…Mukai, T. (1997). New p57KIP2 mutations in Beckwith-Wiedemann syndrome. *Hum Genet*, *100*(5-6), 681-683. <https://doi.org/10.1007/s004390050573>

Hatada, I., Ohashi, H., Fukushima, Y., Kaneko, Y., Inoue, M., Komoto, Y.,…Mukai, T. (1996). An imprinted gene p57KIP2 is mutated in Beckwith-Wiedemann syndrome. *Nat Genet*, *14*(2), 171-173. <https://doi.org/10.1038/ng1096-171>

Jurkiewicz, D., Skórka, A., Ciara, E., Kugaudo, M., Pelc, M., Chrzanowska, K., & Krajewska-Walasek, M. (2020). Rare clinical findings in three sporadic cases of Beckwith-Wiedemann syndrome due to novel mutations in the CDKN1C gene. *Clin Dysmorphol*, *29*(1), 28-34. <https://doi.org/10.1097/mcd.0000000000000307>

Lam, W. W., Hatada, I., Ohishi, S., Mukai, T., Joyce, J. A., Cole, T. R.,…Maher, E. R. (1999). Analysis of germline CDKN1C (p57KIP2) mutations in familial and sporadic Beckwith-Wiedemann syndrome (BWS) provides a novel genotype-phenotype correlation. *J Med Genet*, *36*(7), 518-523.

Lee, M. P., DeBaun, M., Randhawa, G., Reichard, B. A., Elledge, S. J., & Feinberg, A. P. (1997). Low frequency of p57KIP2 mutation in Beckwith-Wiedemann syndrome. *Am J Hum Genet*, *61*(2), 304-309. <https://doi.org/10.1086/514858>

Li, M., Squire, J., Shuman, C., Fei, Y. L., Atkin, J., Pauli, R.,…Weksberg, R. (2001). Imprinting status of 11p15 genes in Beckwith-Wiedemann syndrome patients with CDKN1C mutations. *Genomics*, *74*(3), 370-376. <https://doi.org/10.1006/geno.2001.6549>

O'Keefe, D., Dao, D., Zhao, L., Sanderson, R., Warburton, D., Weiss, L.,…Tycko, B. (1997). Coding mutations in p57KIP2 are present in some cases of Beckwith-Wiedemann syndrome but are rare or absent in Wilms tumors. *Am J Hum Genet*, *61*(2), 295-303. <https://doi.org/10.1086/514854>

Otsuji, S., Nishio, Y., Tsujita, M., Rio, M., Huber, C., Antón-Plágaro, C.,…Kato, K. (2022). Clinical diversity and molecular mechanism of VPS35L-associated Ritscher-Schinzel syndrome. *J Med Genet*. <https://doi.org/10.1136/jmg-2022-108602>

Percesepe, A., Bertucci, E., Ferrari, P., Lugli, L., Ferrari, F., Mazza, V., & Forabosco, A. (2008). Familial Beckwith-Wiedemann syndrome due to CDKN1C mutation manifesting with recurring omphalocele. *Prenat Diagn*, *28*(5), 447-449. <https://doi.org/10.1002/pd.1991>

Romanelli, V., Belinchón, A., Benito-Sanz, S., Martínez-Glez, V., Gracia-Bouthelier, R., Heath, K. E.,…Lapunzina, P. (2010). CDKN1C (p57(Kip2)) analysis in Beckwith-Wiedemann syndrome (BWS) patients: Genotype-phenotype correlations, novel mutations, and polymorphisms. *Am J Med Genet A*, *152a*(6), 1390-1397. <https://doi.org/10.1002/ajmg.a.33453>

Sparago, A., Cerrato, F., Pignata, L., Cammarata-Scalisi, F., Garavelli, L., Piscopo, C.,…Riccio, A. (2021). Variable Expressivity of the Beckwith-Wiedemann Syndrome in Four Pedigrees Segregating Loss-of-Function Variants of CDKN1C. *Genes (Basel)*, *12*(5). <https://doi.org/10.3390/genes12050706>

Welsh, H. I., Stockley, T. L., Parkinson, N., & Ardinger, H. H. (2012). CDKN1C mutations and genital anomalies. *Am J Med Genet A*, *158a*(1), 265. <https://doi.org/10.1002/ajmg.a.34388>

Yatsuki, H., Higashimoto, K., Jozaki, K., Koide, K., Okada, J., Watanabe, Y.,…Soejima, H. (2013). Novel mutations of CDKN1C in Japanese patients with Beckwith-Wiedemann syndrome. *Genes & Genomics*, *35*. <https://doi.org/10.1007/s13258-013-0079-3>
